# How large are the consequences of covariate imbalance in cluster randomized trials: a simulation study with a continuous outcome and a binary covariate at the cluster level

**DOI:** 10.1186/s12874-016-0182-7

**Published:** 2016-07-11

**Authors:** Mirjam Moerbeek, Sander van Schie

**Affiliations:** Department of Methodology and Statistics, Utrecht University, P.O. Box 80140, 3508 TC Utrecht, The Netherlands

**Keywords:** Cluster randomization, Covariate imbalance, Unadjusted linear mixed model, Adjusted linear mixed model, Simulation study

## Abstract

**Background:**

The number of clusters in a cluster randomized trial is often low. It is therefore likely random assignment of clusters to treatment conditions results in covariate imbalance. There are no studies that quantify the consequences of covariate imbalance in cluster randomized trials on parameter and standard error bias and on power to detect treatment effects.

**Methods:**

The consequences of covariance imbalance in unadjusted and adjusted linear mixed models are investigated by means of a simulation study. The factors in this study are the degree of imbalance, the covariate effect size, the cluster size and the intraclass correlation coefficient. The covariate is binary and measured at the cluster level; the outcome is continuous and measured at the individual level.

**Results:**

The results show covariate imbalance results in negligible parameter bias and small standard error bias in adjusted linear mixed models. Ignoring the possibility of covariate imbalance while calculating the sample size at the cluster level may result in a loss in power of at most 25 % in the adjusted linear mixed model. The results are more severe for the unadjusted linear mixed model: parameter biases up to 100 % and standard error biases up to 200 % may be observed. Power levels based on the unadjusted linear mixed model are often too low. The consequences are most severe for large clusters and/or small intraclass correlation coefficients since then the required number of clusters to achieve a desired power level is smallest.

**Conclusions:**

The possibility of covariate imbalance should be taken into account while calculating the sample size of a cluster randomized trial. Otherwise more sophisticated methods to randomize clusters to treatments should be used, such as stratification or balance algorithms. All relevant covariates should be carefully identified, be actually measured and included in the statistical model to avoid severe levels of parameter and standard error bias and insufficient power levels.

## Background

Randomized controlled trials are considered the gold standard for evaluating the effect of a new treatment relative to an old treatment, a placebo or no treatment at all. A necessary (but not sufficient) condition for the estimate of the treatment effect to be unbiased is that subjects in a given treatment group are not contaminated by those in another. This condition is often not fulfilled in trials that are offered in naturally existing groups, such as schools, general practices or communities, especially so when the treatments rely on interpersonal communication in risk reduction sessions and peer pressure groups [1]. For this reason, cluster randomization is often preferred above individual randomization. With cluster randomized trials complete clusters are randomized to treatment conditions and all subjects within a cluster receive the same treatment. So, cluster randomized trials may reduce logistical and administrative costs. Community interventions, such as mass media interventions using TV, are directed towards the whole cluster and cannot be delivered at the individual level. In such trials there is no alternative to cluster randomization. Cluster randomization has become popular in the medical, health and behavioural sciences over the past decades and relevant textbooks on this research design are [2–6].

The number of clusters in a cluster randomized trial is often low [7]. As a consequence, random assignment of clusters to treatment conditions does not ensure the treatment groups are comparable at baseline with respect to all variables at the subject and cluster level that have an effect on the outcome variable. In other words, it is likely there is covariance imbalance at baseline. It is therefore good practice to identify such variables based on findings in the literature or experts’ expectations, to actually measure them and to include them as covariates in an adjusted linear mixed model. As such, an unbiased estimate of the effect of treatment relative to the control can be obtained. In addition to that, part of the residual variance at the cluster and/or subject level is explained by the covariate, which has an advantageous effect on precision and power [8], especially so when the covariate has a strong effect on the outcome. The drawback, however, is that this comes at the cost of degrees of freedom, which may have a decreasing effect on the power of the test on treatment effect, especially when the number of clusters is small. Furthermore, it is known that less efficient estimates of the treatment effect, and hence lower power levels, are obtained with larger degrees of covariate imbalance [9, 10]. Finally, covariate imbalance should not only be taken into account while analysing the data but also while performing an a priori power analysis to calculate the required number of clusters to achieve a desired power level. Ignoring the possibility of covariate imbalance results in an underestimate of the required number of clusters and hence insufficient power, even when the covariates are adjusted for in an adjusted linear mixed model.

In practice it often occurs not all relevant covariates are known and/or not all of them have been measured. As a consequence, type I or type II error rates may be inflated. The degree of inflation depends on the precision of the treatment effect estimator, which is affected by the strength of the covariate effect and degree of covariate imbalance. A recent study quantified the effect of covariate imbalance on bias and precision of the treatment effect estimator and the power of the test on treatment effect for simple randomized trials [11]. With such trials subjects are randomly assigned to treatment conditions and there is no nesting of subjects within clusters. One continuous covariate was considered and the adjusted linear model was compared to the unadjusted linear model, which does not include the covariate. The conclusion was that, while the adjusted linear model produces unbiased estimates of the treatment effect, the unadjusted linear model is subject to bias. The unadjusted linear model was found to be less precise than the adjusted linear model. Finally it was found that the power of the unadjusted linear model can be much larger than the anticipated level at certain levels of imbalance.

The number of clusters in a cluster randomized trial is typically much lower than the number of subjects in a simple randomized trial, hence covariance imbalance is more likely to occur in cluster randomized trials than in simple randomized trials. For this reason it is worthwhile to extend the study in reference [11] to cluster randomized trials. We study to what extent a loss of power is achieved when the power calculation is done under the assumption of covariate balance, while the randomization process results in covariate imbalance. In other words, we study the loss of power when the unadjusted linear mixed model is assumed in an a priori power analysis but the adjusted linear mixed model is used for data analysis. In addition to that, we investigate the effects of ignoring relevant covariates while analysing the data. Stated differently, we study the size of the parameter and standard error bias when the unadjusted linear mixed model is used to analyse the data while the data are generated by an adjusted linear mixed model. Also, we study the power levels achieved with the unadjusted linear mixed model.

The focus is on a covariate at the cluster-level. Such a covariate may be a cluster-level characteristic, such as school-type (e.g. public versus private) in school-based intervention studies, or an aggregate, such as a school’s mean socio-economic status. Associations between the covariate and outcome tend to be much stronger between than within clusters [12, 13]. Furthermore, it is often less expensive and less time-consuming to measure cluster-level covariates than subject-level covariates, simply because the number of clusters is always (much) smaller than the number of subjects.

The data obtained from cluster randomized trials have a multilevel data structure with subjects nested within clusters. Such data have to be analysed by using the mixed model. Ignoring the multilevel data structure results in too liberal tests with respect to the effect of treatment [14]. Our approach to study the effects of covariate imbalance is a simulation study in which various sizes of the covariate effect and degrees of covariance imbalance are considered. Cluster size and the intraclass correlation coefficient (i.e. the proportion variance at the cluster level) are known to have an effect on precision and power in cluster randomized trials [15], hence various values of these two factors are considered as well.

### Mixed model for cluster randomized trials

To put things in context a school-based intervention study to evaluate the effect of a new type of smoking prevention intervention on smoking behaviour of secondary school students is used as an example. Secondary schools are randomly assigned to treatment conditions and all students within a school receive the same condition. The covariate we consider is educational level; it is binary and measured at the level of the secondary school: vocational schools train their students for specific vocations while high schools prepare their students for follow-up education.

The outcome we consider is continuous with higher scores related to higher levels of smoking abstinence. It is very likely scores of students within the same school are correlated, for instance as a result of mutual influence and shared norms towards smoking. Ignoring such correlation results in overoptimistic conclusions with respect to the effect of intervention [14]. To account for such dependencies the adjusted linear mixed model should be used. The score *y*_*ij*_ of student *i* in school *j* is given by

1 $$ {y}_{ij}={\beta}_0+{\beta}_1{x}_j+{\beta}_2{z}_j+{u}_j+{e}_{ij}. $$

Treatment condition is denoted *x*_*j*_ and has the value 0 for the control and 1 for the intervention. The covariate is denoted *z*_*j*_ and has the value 0 for vocational schools and 1 for high schools. Note that both *x*_*j*_ and *z*_*j*_ have a subscript *j* but not *i* since they vary between but not within schools. The regression weights *β*_0_, *β*_1_ and *β*_2_ are the intercept and the effect of the intervention and covariate, respectively. The random school-level effect *u*_*j*_ is the discrepancy of the mean in school *j* and the mean score in its treatment condition and school type, and the random subject-level error term *e*_*ij*_ is the discrepancy of the score of student *i* and the mean score of its school *j*. Both random effects are assumed to be independent of each other and to follow a normal distribution with zero mean and variances *σ*_*u*_^2^ and *σ*_*e*_^2^. The total error variance *σ*^2^ = *σ*_*u*_^2^ + *σ*_*e*_^2^ is the sum of these two variance components.

On the basis of the adjusted linear mixed model (1) the treatment effect is the difference in expected outcomes across the two treatments, corrected for the covariate:

2 $$ {\beta}_1={\mu}_{y_I}-{\mu}_{y_C}-{\beta}_2\left({\mu}_{z_I}-{\mu}_{z_C}\right). $$

Here, ${\mu}_{y_I}$ and ${\mu}_{y_C}$ are the expected outcome scores in the intervention and control condition, and ${\mu}_{z_I}$ and ${\mu}_{z_C}$ are the expected covariate scores in the intervention and control conditions. The expectations *μ* can be replaced by their observed means as calculated from a sample to obtain an estimate of the treatment effect. The standard error of this estimator is

3 $$ se\left({\hat{\beta}}_1\right)=\sqrt{\frac{\sigma_e^2+{n}_1{\sigma}_u^2}{n_1{n}_2\left(1-{\rho}_{xz}^2\right)}}, $$

with *n*_1_ and *n*_2_ the common school size and number of schools, and *ρ*_*xz*_ the correlation between treatment condition and the covariate. The larger this correlation, the larger the degree of covariate imbalance. Eq. (3) shows that the standard error se $\left({\hat{\beta}}_1\right)$ becomes larger when ${\rho}_{xz}^2$ increases. In other words, covariate imbalance results in a loss of efficiency. The factor 1/($1-{\rho}_{xz}^2$) is the variance inflation factor and it is the same equation as for designs without nesting of subjects in clusters [9]. The standard error (3) is estimated by replacing the variance components and the correlation between treatment and the covariate by their estimates ${\hat{\sigma}}_e^2$ and ${\hat{\sigma}}_u^2$ and *r*_*xz*_.

The test statistic for the test on treatment effect is calculated as $t={\hat{\beta}}_1/\mathrm{s}\mathrm{e}\left({\hat{\beta}}_1\right)$ and follows a central *t* distribution with *n*_2_ − 3 degrees of freedom under the null hypothesis of no treatment effect *H*_0_ : *β*_1_ = 0. The statistical power is the probability of rejecting the null hypothesis when it is false. The number of clusters to achieve a power level of at least 1 − *β* in a test with a two-sided alternative hypothesis and type I error rate *α* is calculated as

4 $$ {n}_2=4\frac{\sigma_e^2+{n}_1{\sigma}_u^2}{n_1\left(1-{\rho}_{xz}^2\right)}{\left(\frac{z_{1-\alpha /2}+{z}_{1-\beta }}{\beta_1}\right)}^2. $$

This will always give a non-integer value which has to be rounded upwards to the nearest integer to achieve the desired power level.

In practice it often occurs not all relevant covariates are measured, hence they cannot be included into the regression model. The unadjusted linear mixed model ignores the covariate *z*_*j*_:

5 $$ {y}_{ij}={\beta}_0^{*}+{\beta}_1^{*}{x}_j+{u}_j^{*}+{e}_{ij}^{*}. $$

The model parameters are now indicated by an asterisk to distinguish them from their counterparts in the adjusted linear mixed model (1). The error variances also have an asterisk: *σ*_e_^* 2^ and *σ*_u_^* 2^ and again the errors are assumed normally distributed with zero mean. As *z*_*j*_ varies between but not within schools, it only explains part of the error variance at the between-school level, hence *σ*_e_^* 2^ = *σ*_*e*_^2^, *σ*_u_^* 2^ > *σ*_*u*_^2^ and *σ*_u_^* 2^ + *σ*_e_^* 2^ > *σ*_*u*_^2^ + *σ*_*e*_^2^.

On the basis of the unadjusted linear mixed model the treatment effect is the difference in expected outcomes across the treatments:

6 $$ {\beta}_1^{*}={\mu}_{y_I}-{\mu}_{y_C} $$

Here, ${\mu}_{y_I}$ and ${\mu}_{y_C}$ are the expected outcomes in the intervention and control condition. Replacing the expectations *μ* by their observed means as calculated from a sample provides an estimate ${\hat{\beta}}_1^{*}$ of the treatment effect. A comparison of Eqs. (2) and (6) shows that ignoring the covariate results in a biased estimate of the effect of treatment, unless the covariate has no effect on the outcome (i.e. *β*_2_ = 0) or it is balanced across treatment conditions (i.e. ${\mu}_{z_I}={\mu}_{z_C}$). The standard error of the estimator ${\hat{\beta}}_1^{*}$ is

7 $$ se\left({\hat{\beta}}_1^{*}\right)=\sqrt{\frac{\sigma_e^{*2}+{n}_1{\sigma}_u^{*2}}{n_1{n}_2}} $$

and it is estimated by replacing the variance components by their estimates ${\hat{\sigma}}_e^{*2}$ and ${\hat{\sigma}}_u^{*2}$. The test statistic for the test on treatment effect is calculated as $t={\hat{\beta}}_1^{*}/se\left({\hat{\beta}}_1^{*}\right)$ and follows a central *t* distribution with *n*_2_ − 2 degrees of freedom under the null hypothesis of no treatment effect *H*_0_ : *β*_1_^*^ = 0. Note that there is one more degree of freedom than in the adjusted linear mixed model since the effect of the covariate is not estimated.

For simplicity it is assumed that half of the schools are vocational schools and the others are high schools. Furthermore, half of the schools are randomly assigned to receive the intervention, which implies the other half receives the control. Let us assume the intervention results in higher outcome scores (i.e. *β*_1_ > 0) and that higher outcome scores are observed in high schools than in vocational schools (i.e. *β*_2_ > 0). Let us define positive imbalance as a situation where, by chance, the random assignment results in more than half of the schools in the intervention condition being high schools. This would imply ignoring the covariate in the statistical model results in an overestimated treatment effect. Negative imbalance is the opposite situation and the treatment effect is underestimated.

The number of high schools in the intervention condition follows from a hypergeometric distribution with parameters *n*_2_ (the total number of schools), $\frac{1}{2}{n}_2$ (the number of high schools) and $\frac{1}{2}{n}_2$ (the number of schools that are randomized to receive the intervention). The expectation of the hypergeometric distribution is $\frac{1}{4}{n}_2$, which implies that both treatment groups consist of as many high schools as vocational schools. The degree of covariate imbalance is expressed by quantiles from the hypergeometric distribution rather than the percentage high schools in the intervention condition. As such we adopt the approach by Egbewale, Lewis and Sim [11] to express the degree of covariate imbalance. The percentage high schools in the intervention condition for a given quantile depends on the total number of schools. For the 95^th^ quantile, for instance, this percentage is equal to 70 when the total number of schools is 20, and it is equal to 56 when the total number of schools is 200. This illustrates large percentage deviations from the expectation are more likely to occur for a small number of clusters than for a large number of clusters.

## Methods

A simulation study was conducted to gain further insight in the size of the effects of covariate imbalance in cluster randomized trials. Data generation and parameter estimation were done in R [16]. Parameter estimation was done using the function lme from the package nlme for linear and non-linear mixed effect models [17]. Estimation was done by means of restricted maximum likelihood since it produces less biased estimates of the variance components than full information maximum likelihood, especially when the number of clusters is small.

Four factors are considered in the simulation study: cluster size, intraclass correlation coefficient, covariate effect size and degree of covariate imbalance. For cluster size the values 5, 30 and 50 were used. These values are common in respectively households, school classes and departments in hospitals or companies, and were also used by Maas and Hox [18]. Note that the cluster size did not vary across clusters; the implication of varying cluster sizes is discussed later on. The intraclass correlation coefficient *ρ* measures the proportion of the variance that is located at the cluster level. It is calculated as *ρ* = *σ*_*u*_^2^/(*σ*_*u*_^2^ + *σ*_*e*_^2^), and the values 0.01, 0.05, and 0.1 were used in this simulation study. These values cover a wide range of plausible values, with small values common in large clusters, such as hospitals, and large values common in small clusters, such as households. Without loss of generality the total error variance was fixed to *σ*^2^ = *σ*_*u*_^2^ + *σ*_*e*_^2^ = 1. The covariate effect size *β*_2_ had levels 0.2, 0.5, and 0.8, which correspond to small, medium and large effect sizes [19]. We do not consider the case where the covariate has no effect (i.e. *β*_2_ = 0) because the consequences of ignoring covariate imbalance are expected to be negligible. The degree of covariate imbalance is expressed as quantiles of the hypergeometric distribution and the following values were used: 0.025, 0.05, 0.1, 0.5, 0.9, 0.95, and 0.975. Values above 0.5 imply positive imbalance (i.e. too many high schools in the intervention condition). The value 0.5 implies perfect balance of the covariate across the treatments and is also considered.

In total 3*3*3*7 = 189 combinations of factor levels were considered and these are called simulation conditions henceforward. For each simulation condition 5000 data sets were generated from the adjusted linear mixed model and analysed by means of the adjusted linear mixed model and unadjusted linear mixed model. For each combination of the cluster size and intraclass correlation coefficient the required number of clusters to achieve a power level 1 − *β* = 0.8 to detect a medium treatment effect *β*_1_ = 0.5 in a test with a two-sided alternative hypothesis and *α* = 0.05 was calculated from (4). This gives a non-integer value which is rounded upwards such that an even number of clusters is available per treatment condition. Only when an even number of clusters per treatment is used balance of the binary covariate at the cluster level can be achieved. This is important because we want to include perfect balance of the covariate across the treatments in our simulation study (see above). Due to rounding upwards to integer values the nominal power levels were somewhat larger than 0.8, and they also varied across the simulation conditions. In practice the possibility of covariate imbalance is often ignored while calculating the required number of clusters. The required number of clusters is therefore calculated under the assumption of covariate balance (i.e. *ρ*_*xz*_ = 0), but data are generated under various degrees of imbalance and covariate effect size to study the effect of covariate imbalance on parameter estimates and empirical power.

Various criteria were used to evaluate the effects of covariate imbalance. The primary focus was on the treatment effect, since this is the parameter of main interest in intervention research. The percentage parameter bias is defined as

8 $$ parameter\  bias=100\%*\frac{\overline{{\hat{\beta}}_1}-{\beta}_1}{\beta_1}, $$

where $\overline{{\hat{\beta}}_1}$ is the estimated treatment effect averaged across the 5000 generated data sets and the population value of *β*_1_ is 0.5 in this simulation study. For the unadjusted linear mixed model $\overline{{\hat{\beta}}_1}$ is replaced by $\overline{{\hat{\beta}}_1^{*}}$. Positive biases indicate the treatment effect is overestimated, which may result in too liberal tests on treatment effect and hence inflated type I error rates. Acceptable biases are below 10 % in absolute value [20].

The second criterion for evaluation is the percentage standard error bias:

9 $$ standard\  error\  bias=100\%*\frac{\overline{se\left({\hat{\beta}}_1\right)}-sd\left({\hat{\beta}}_1\right)}{sd\left({\hat{\beta}}_1\right)}. $$

Here the mean standard error of the treatment effect estimates is compared to the standard deviation of the 5000 treatment effect estimates. Positive bias implies the standard errors are overestimated, which may result in a loss of power. Acceptable biases are below 5 % in absolute value [20].

The third criterion is the empirical power level. For each simulation condition it is approximated as the percentage of data sets for which the null hypothesis of no treatment effect was rejected in a test with a two-sided alternative hypothesis and type I error rate *α* = 0.05.

## Results

**Fig. 1:**
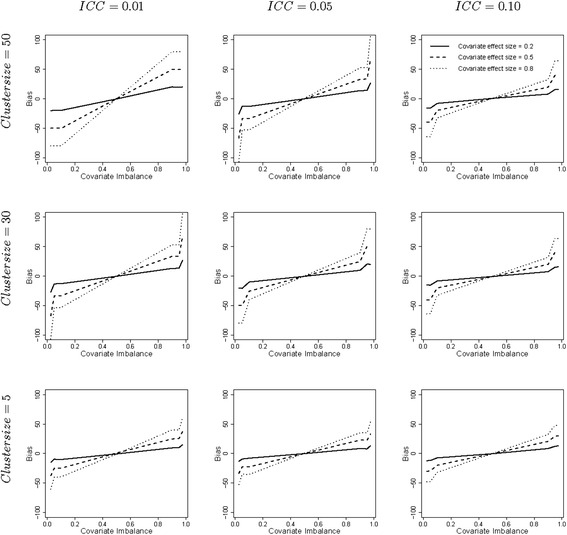
Parameter bias for the linear mixed model. Legend: The degree of covariate imbalance is given on the horizontal axes and expressed as quantiles of the hypergeometric distribution. Values below 0.5 imply negative covariate imbalance, values above 0.5 imply positive covariate imbalance and the value 0.5 implies covariate balance

For all simulation conditions the parameter bias of the adjusted linear mixed model was negligible: the largest bias was −1.1 % (figures not shown). For the unadjusted linear mixed model the parameter biases are much larger because it ignores the covariate and in the worst cases these biases are slightly over 100 %, as is depicted in Fig. 1. The lines cut when the covariate is balanced across treatments (i.e. when covariate imbalance = 0.5). For negative covariate imbalance the bias is negative, and vice versa for positive covariate imbalance. In terms of the example: the effect of treatment is underestimated when there are too many vocational schools in the intervention condition, and overestimated when there are too many high schools in the intervention condition. In general the biases increase with increasing degree of covariate imbalance and covariate effect size. This also follows from a comparison of Eqs. (2) and (6). The bias is ${\beta}_2\left({\mu}_{z_I}-{\mu}_{z_C}\right)$ and increases with covariate effect size *β*_2_ and the difference between expected covariate scores ${\mu}_{z_I}$ and ${\mu}_{z_C}$ in both treatment conditions. Furthermore, the bias increases with increasing cluster size. The effect of covariate imbalance becomes slightly less pronounced when the intraclass correlation increases.

**Fig. 2:**
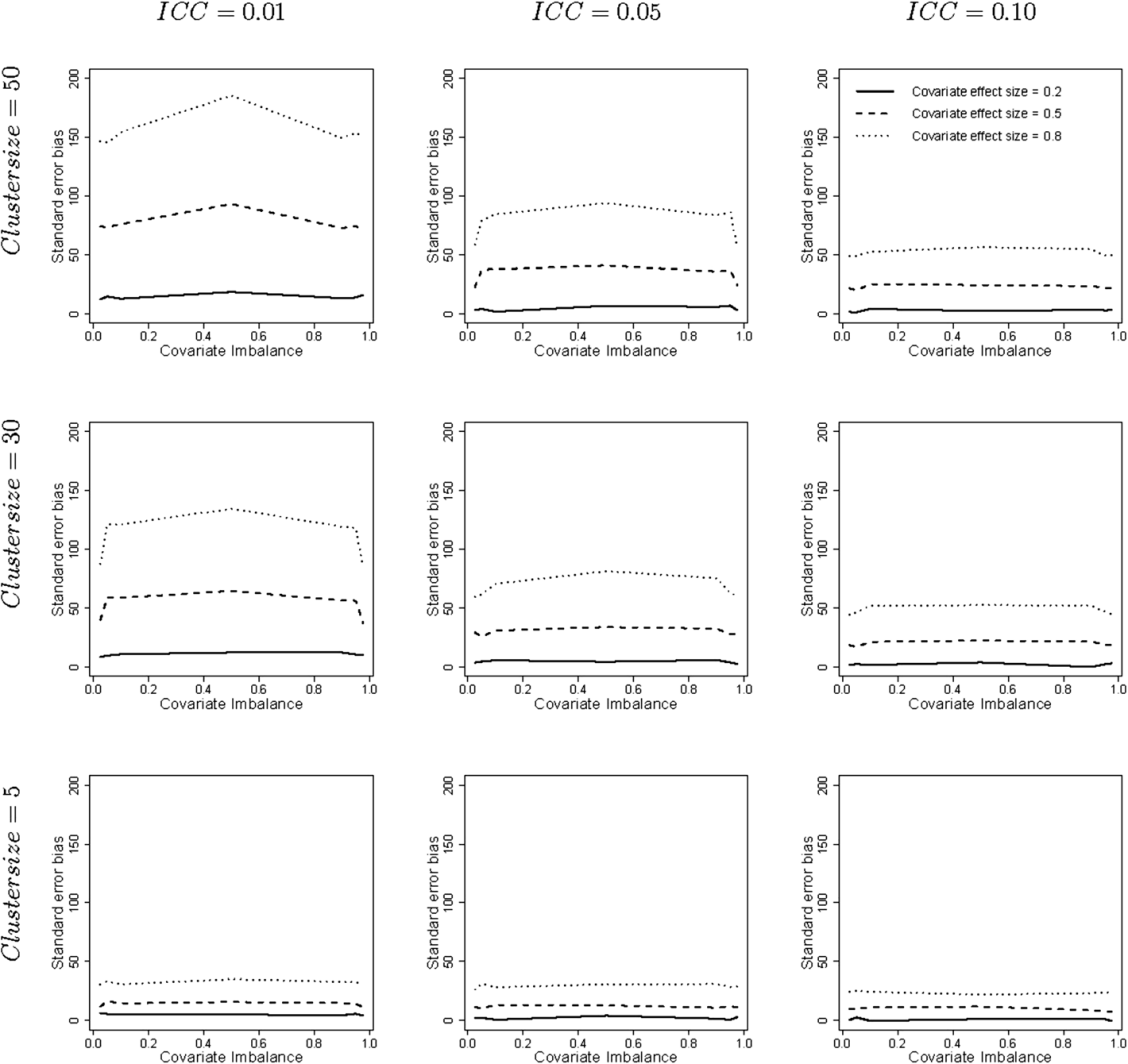
Standard error bias for the linear mixed model. Legend: The degree of covariate imbalance is given on the horizontal axes and expressed as quantiles of the hypergeometric distribution. Values below 0.5 imply negative covariate imbalance, values above 0.5 imply positive covariate imbalance and the value 0.5 implies covariate balance

For the adjusted linear mixed model the standard error biases were small and the largest bias was −5.1 % (figures not shown). The standard error biases are much larger for the unadjusted linear mixed model and up to almost 200 %, see Fig. 2. The standard error bias is always positive since the unadjusted linear mixed model overestimates the variance component at the cluster level, and hence it overestimates the standard error of the treatment effect estimate (see Eq. (7)). The standard error bias increases with increasing covariate effect size and increasing cluster size. These effects were also found for the parameter bias. For large cluster size *n*_1_ = 50 the standard error bias decreases with increasing intraclass correlation coefficient but this effect becomes weaker when the cluster size decreases. Surprisingly the standard error bias is most often largest when the covariate is uncorrelated with treatment.

**Fig. 3:**
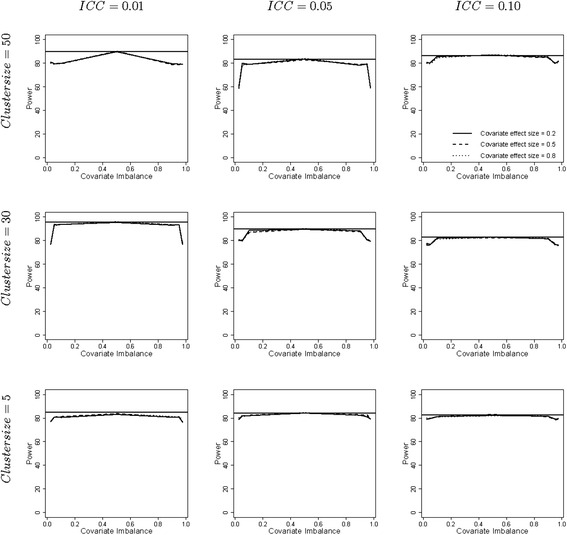
Empirical power levels for the adjusted linear mixed model. Legend: The degree of covariate imbalance is given on the horizontal axes and expressed as quantiles of the hypergeometric distribution. Values below 0.5 imply negative covariate imbalance, values above 0.5 imply positive covariate imbalance and the value 0.5 implies covariate balance. The horizontal solid line represents the nominal power level

Figure 3 shows the empirical power levels as achieved by the adjusted linear mixed model. Again note that in this simulation study the number of clusters to achieve 80 % power was calculated under the assumption of covariate balance while the data were generated for various levels of covariate imbalance. The number of clusters as calculated from Eq. (4) is often a non-integer and has to be rounded upwards to ensure a power of at least 80 %. As a consequence the nominal power is somewhat above 80 %, especially in the cases where the cluster size is large and/or the intraclass correlation coefficient is small. The nominal power is shown by the horizontal line within each subplot. The largest empirical power levels are observed in case of covariate balance and these are very close to the nominal power levels. Power decreases as the degree of covariate imbalance increases because the standard error of the treatment effect increases with increasing degree of covariate imbalance (see Eq. (3)). This decrease is largest for large cluster size and small intraclass correlation coefficient. In the worst case a loss of power of almost 25 % is observed. The covariate effect size has a negligible effect on empirical power levels.

**Fig. 4:**
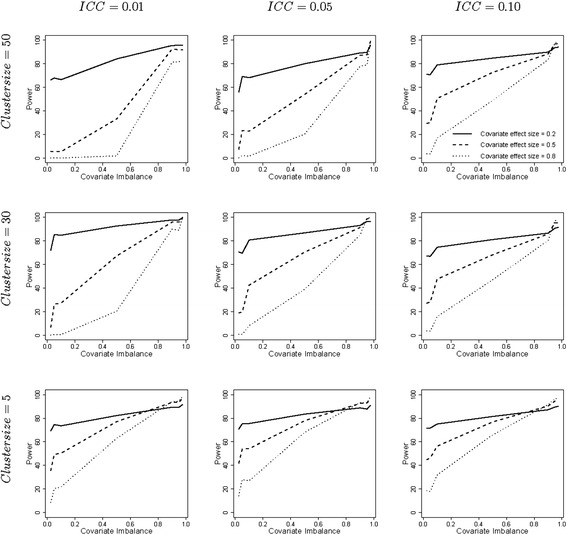
Empirical power levels for the linear mixed model. Legend: The degree of covariate imbalance is given on the horizontal axes and expressed as quantiles of the hypergeometric distribution. Values below 0.5 imply negative covariate imbalance, values above 0.5 imply positive covariate imbalance and the value 0.5 implies covariate balance

Figure 4 shows the power levels for the unadjusted linear mixed model are much different from those for the adjusted linear mixed model. For negative imbalance the power can be much below the 80 % level. This can be explained by an underestimation of the treatment effect (see Fig. 1) and an overestimation of the related standard error (see Fig. 2). In these cases the largest power is achieved for the smallest covariate effect size. For large values of positive imbalance, the overestimate of the treatment effect compensates the overestimate of the related standard error and the power level is above 80 %. Furthermore it should be noted that an absence of covariate imbalance does not imply the power level is 80 %. Finally, it should be noted that, as in the previous figures, the strongest effects of covariate imbalance are generally observed for large cluster size and small intraclass correlation coefficient.

## Discussion

In this simulation study we focussed on cluster randomized trials with a continuous outcome at the individual level and a binary outcome at the cluster level. Our findings are in accordance with those for simple randomized trials with a continuous outcome [11]. Covariate imbalance has a negligible effect on parameter bias and a small effect on standard error bias when the adjusted linear mixed model is used. Ignoring covariate balance while calculating the required number of clusters may result in a loss of power up to 25 % for the adjusted linear mixed model. It is therefore good practice to account for covariate imbalance while calculating the number of clusters or to use more sophisticated methods to randomize clusters to treatment conditions, for instance stratification or balance algorithms, such as the one in [21].

Covariate imbalance has much more severe consequences when the covariate is ignored while analysing the data. For the unadjusted linear mixed model the treatment effect was overestimated or underestimated by at most 100 % and its standard error was overestimated by at most 200 % in our simulation study. In most cases the power levels were below 80 % but for very large levels of positive imbalance they were above 80 %. This stresses the need to carefully consider which covariates are important in the study at hand, to actually measure them and to include them in the statistical model. A new tool based on propensity scores is useful to detect covariate imbalances [22].

For both models and for any of the three criteria of evaluation it was observed that the strongest results occurred when the cluster size was large and the intraclass correlation coefficient was small. This is not surprising because in these cases the number of clusters to achieve 80 % power is smallest. The smaller the number of clusters the more likely the percentage high schools in the intervention condition deviates from 50 %. So one should take extra care in trials with large clusters and small degree of dependence among the subjects within a cluster. For instance, covariate imbalance can result in more severe consequences when the cluster randomized trial has general practices as unit of randomization rather than households.

In our simulation we assumed all clusters were of equal size. In practice cluster sizes vary and this can be compensated by adding 11 % more clusters [23]. As we argued above, a larger number of clusters results in weaker effects of cluster imbalance.

We acknowledge the setup of our simulation study is somewhat limited. We considered a continuous outcome variable, a single binary covariate at the cluster level, both levels of the covariate were equally represented, and both treatment groups were of equal size. It would also be interesting to consider an individual-level covariate, such as a pre-treatment score or a socio-demographic variable. We expect the potential implications of imbalance of an individual-level covariate to depend on the between- and within-level variability of such a covariate. The higher the between-level variability as compared to the within-level variability, the more the implications of covariate imbalance will correspond to the implications of covariate imbalance of a covariate at cluster level. On the other extreme, covariate imbalance cannot occur when the individual-level covariate only varies within clusters but not between clusters because treatment condition is a cluster level variable. In that case both the unadjusted linear mixed model and adjusted linear mixed model will yield an unbiased estimate of the treatment effect size, but the associated standard error will be smaller for the adjusted linear mixed model because part of the within-cluster variance is explained by the covariate. The implications of imbalance of an individual-level covariate that varies both between and within clusters will be between these two extremes and will have to be studied in future research. Future research could also focus on trials with more covariates which can be measured at the subject and/or cluster level and which can be measured as categorical or scale variables. In any case we expect a loss of power in adjusted linear mixed models and parameter and standard error biases in unadjusted linear mixed models. As for now, it remains unclear how large the effects may be.

## Conclusion

We can conclude that covariate imbalance may result in a loss of power in adjusted linear mixed models. It is therefore advocated to be aware of the possibility of covariate imbalance while calculating the number of clusters, or to apply stratified randomization or balance algorithms. In addition, ignoring relevant covariates while analysing the data may lead to severely biased estimates of the treatment effect and its standard error. It is therefore important all relevant covariates are identified, measured and included in the regression model. This recommendation holds for simple randomized trials, but is even more important for cluster randomized trials because for this type of trials the number of units to be randomized is often low.
